# Implementation of a uniform nationwide medical licensing examination in general practice. A feasibility study

**DOI:** 10.3205/zma001492

**Published:** 2021-06-15

**Authors:** Iris Demmer, L. Selgert, A. Altiner, E. Baum, A. Becker, L. Schmittdiel, I. Streitlein-Böhme, M. Michiels-Corsten, S. Zutz, E. Hummers, J. Jünger

**Affiliations:** 1 University Medical Center Göttingen, Department of General Practice, Göttingen, Germany; 2 German National Institute for state examinations in Medicine, Pharmacy and Psychotherapy (IMPP), Mainz, Germany; 3 University Medical Center Rostock, Department of General Practice, Rostock, Germany; 4 University of Marburg, Department of General Practice/Family Medicine, Marburg, Germany; 5 General Practice, Munich, Germany; 6 Ruhr-University Bochum, Department of General Practice/Family Medicine, Bochum, Germany; 7 Practice for general and family medicine, Neubukow, Germany

**Keywords:** undergraduate medical education, final year, licensing examination, general medical, outpatient

## Abstract

**Objective: **A competency-based training of medical students that is adapted to the realities of care is required internationally and is being intended in Germany with the Master Plan for Medical Studies 2020.

In order to test these competencies, the German National Institute for state examinations in Medicine, Pharmacy and Psychotherapy (IMPP) has developed a concept for the redesign of the final part of the medical licensing examination in Germany. It focuses on general and interprofessional healthcare in the examination with outpatients. The aim of this work is to assess the feasibility of the new final examination on the basis of pilot examinations in family practices and to derive further steps for the national implementation.

**Methods: **Fourteen medical students in their internship year completed a full examination with patients aged 42 to 84 years. Examiners evaluated the examination performance using standardised evaluation forms. Feasibility was qualitatively assessed in terms of compliance with content and time limits, examination results, patient reflections, and implementation in the practice.

**Results: **Students were able to complete all tasks within the given time frame. Based on the evaluation forms, the examiners assessed the performance of the students. Patients appreciated the structured course of the examination in the familiar location of their family practice.

For the nationwide implementation of the examination, 2,500 examination practices are required for about 10,000 examinees per year. Four students can then be examined on two days per year in each practice.

**Conclusions: **Oral-practical examinations with outpatients in general medical practices can be carried out successfully throughout the nation. An implementation of the examinations throughout Germany requires that medical studies are restructured and that this new curriculum is implemented as intended by the Master Plan for Medical Studies 2020. Furthermore, training and remuneration of examiners together with a legal framework for the new examination must be established.

## Zusammenfassung

**Zielsetzung: **Eine kompetenzbasierte, an der Versorgungsrealität orientierte Ausbildung Medizinstudierender wird international gefordert und in Deutschland mit dem Masterplan Medizinstudium 2020 operationalisiert.

Zur Prüfung dieser Kompetenzen hat das IMPP ein Konzept zur Neugestaltung der medizinischen Abschlussprüfung erstellt und fokussiert dabei mit der Prüfung mit ambulanten Patienten auf die hausärztliche und interprofessionelle Gesundheitsversorgung. Ziel der Arbeit war es, die Durchführbarkeit der neuen Abschlussprüfung anhand von Pilotprüfungen in allgemeinmedizinischen Lehrpraxen einzuschätzen und daraus weitere Schritte zur nationalen Implementierung abzuleiten.

**Methodik: **Vierzehn Medizinstudierende im Praktischen Jahr absolvierten eine vollständige Prüfung mit Patienten im Alter von 42 bis 84 Jahren. Prüfer beurteilten die Prüfungsleistungen anhand standardisierter Bewertungsbögen. Die Durchführbarkeit wurde qualitativ hinsichtlich Einhaltung inhaltlicher und zeitlicher Vorgaben, Prüfungsergebnissen, Reflexionen der Patienten und Realisierung im Praxisbetrieb eingeschätzt.

**Ergebnisse:** Studierende konnten alle Teilaufgaben im vorgegebenen zeitlichen Rahmen umsetzen. Anhand der Bewertungsbögen konnten ihre Leistungen von den Prüfern beurteilt werden. Patienten wertschätzten den strukturierten Ablauf der Prüfung in dem ihnen vertrauten Prüfungsort Hausarztpraxis. Zur bundesweiten Umsetzung der Prüfung werden für etwa 10.000 Prüflinge pro Jahr 2.500 Prüfpraxen benötigt, in denen insgesamt vier Studierende an zwei Tagen pro Jahr geprüft werden.

**Schlussfolgerung: **Mündlich-praktische Prüfungen mit ambulanten Patienten in allgemeinmedizinischen Praxen sind bundesweit erfolgreich durchführbar. Ihre nationale Implementierung erfordert die Umsetzung der mit dem Masterplan Medizinstudium 2020 beabsichtigten Neustrukturierung des Medizinstudiums, Schulungen und Honorierung der Prüfer und einen rechtlichen Rahmen für die neue Prüfung.

## 1. Background

The majority of patients are cared for in the primary care sector [1]. Medical students are mainly trained on inpatients, however, who represent only a highly selected part of the treatment spectrum [2], [3]. Frenk et al. [4] therefore call for competency-oriented training that is geared to the future reality of healthcare. This requires education systems that prepare students interprofessionally and in a wide range of sectors for future tasks in the medical profession. In this international reform, examinations are given greater importance as the strongest performance-controlling element and should be competency-based as “milestones“ [5], [6]. 

The Federal Ministry of Health and the Federal Ministry of Education and Research in Germany have taken up these challenges and operationalised them with specific measures in the Master Plan for Medical Studies 2020 [7]. The orientation towards competency-based learning and assessment represents a shift in paradigm. With the introduction of a compulsory outpatient quarter in the practical year (PJ), medical students in general medicine can learn the principles of working methods of general practitioners and the care of patients of all ages and with a wide range of concerns. Students can also experience a change of perspective from the university-based maximum care of highly selected patient populations to the primary care level. In this way, mutual collegial understanding between different levels of care can be strengthened in later professional life. 

The aim is to achieve comparability of performance standards at the various faculties through uniformly designed state examinations by the Institute for Medical and Pharmaceutical Examination Questions (IMPP). In order to test the competencies acquired in medical studies, the IMPP has drawn up a concept for redesigning the last part of the medical examination [7], [8]. To date, the oral practical examination in the third part of the Medical Examination (M3) has exclusively taken place at university locations or in teaching hospitals. Here, specialist knowledge is predominantly assessed on inpatients [https://www.gesetze-im-internet.de/_appro_2002/BJNR240500002.html], while medical interviewing is only briefly touched on [9]. The new M3 examination on outpatients will focus on primary care and interprofessional healthcare. It introduces an examination that tests the communicative, practical, and scientific competencies of medical students with real patients at the primary care location. 

The new examination concept for the M3 examination provides an examination based on real patients in the inpatient setting (day one) and in the general practitioner setting (day two). This is followed by an application-related course examination on day three. This concept is depicted in the working draft of the new licensing regulations for physicians (ÄApprO) [10].

Students should be provided with a assessment already during the PJ, with formative feedback using standardised evaluation forms. This is intended to prepare them for the final part of the medical examination as a workplace-based summative examination with a patient in the family practice. The final part focuses on the patients with their needs for appropriate doctor-patient communication, a patient-oriented approach, participation in medical decision-making, and layperson-oriented information about the patient’s health, diseases, and treatment options. 

The aim of our work was to test the oral-practical examination with outpatients in general medical teaching practices and to assess its feasibility (procedure, time required, premises, fairness, reasonableness, and willingness of suitable patients to participate). On the basis of 14 pilot examinations, we examined whether it was possible to implement the examination format designed by the IMPP, including the procedure of the examination with the individual sub-steps, and the evaluation of the examination performance in practice. The next steps for the nationwide implementation of the new examination format are to be derived from the results.

## 2. Methods

### 2.1. Study design

We built on a prototype M3 examination format developed by the IMPP on inpatients [8], [11]. The prototype M3 outpatient examination format was developed in a review process facilitated by IMPP with IMPP staff and the medical school faculty with teaching responsibilities between June 2019 and November 2019. Procedures and evaluation forms were adapted after initial pre-testing in Autumn of 2019 in teaching practices at the University Medical Center Rostock, the University Medical Center Göttingen (UMG), and the Technical University and the Ludwig-Maximilians-University Munich. Subsequently, the pilot study with formative character was carried out on the basis of 14 complete examinations in seven accredited teaching practices (six practices of the UMG and one practice of the University of Marburg) in the period from February to June 2020. For this purpose, positive votes were obtained from the ethics committees of the UMG, the University of Marburg, the medical associations of Lower Saxony and Westphalia-Lippe, and the data protection officers of the UMG. Accident and liability insurances were taken out for the students for the examination matters.

#### 2.2. Examination format

The examination includes eight steps: A comprehensive history taking and complete physical examination of the patient under the supervision of two examiners, an intraprofessional handover to the medical examiners, the creation of a scientific question as a population-intervention-comparison-outcome (PICO) question [12] to be answered by means of an Internet-based literature search, the development of suggestions for outpatient care management, a structured interprofessional handover to a person from another professional group involved in the patient’s healthcare, and finally, the preparation of a report of the physician’s findings and patient-directed report.

#### 2.3. Study participants and study framework

Practices from the PJ teaching practice pools of the UMG and the University of Marburg were informed about the project by the study director and invited to participate. In the formative examination, the PJ teaching physicians acted as the main general medical examiners. The examination step “interprofessional handover” was additionally assessed by a medical assistant from the teaching practice. Co-examiners from the departments of internal medicine, surgery, clinical pharmacology, psychosomatics and psychotherapy, and psychiatry were recruited from the UMG faculty pool and the IMPP. PJ students were personally approached by the study director and asked to participate. 

Participating patients were recruited by the teaching physician of the study practice. Adult patients without significant limitations in communication and without terminal or severe psychiatric illness were eligible for participation. Fourteen Patients aged 42 to 84 years (median: 73 years) provided informed consent for study participation.

#### 2.4. Examination procedure

The students were briefed about the examination procedure from the study director, and were given a written work assignment, access to a computer workstation with Internet access in the examination practice, and electronic templates for writing the reports. 

The examination steps “medical history” and “physical examination” required the patient to be present for approximately one hour. In the subsequent examination steps, phases of written and electronic task-processing by the student alternated with the presentation of partial results in examination discussions (see table 1 (Tab. 1)). Two students were examined alternately on each day of the examination in the practice. The second student started the examination 75 minutes after the first student. Each examination included a 30-minute break and a debriefing with the examination participants.

The main examiner, who was also the primary care physician attending the patient, was released by the patient from their duty of confidentiality to the other study participants. Demographic data were collected from all study participants (all: age, gender; students: semester, M1 and M2 examination results; examiner: highest professional degree, teaching and examination experience; patient: previous experience as an examination patient).

#### 2.5. Evaluation of examination performance using standardised evaluation forms

The examination performances were assessed in each case by the main and co-examiners, and in examination step 5, also by a medical assistant as examiner of another healthcare profession using standardised evaluation forms. Relevant items with anchor criteria were specified, and the items were evaluated on a percentage basis using weighted global rating scales (0-5 points). From the point values achieved in percentage terms, partial grades were created for each examination step that corresponded to the specifications of the currently applicable ÄApprO [https://www.gesetze-im-internet.de/_appro_2002/BJNR240500002.html] for the evaluation of written examinations according to the 60% pass mark. To calculate an overall grade given by each medical examiner, the mean of the partial grades was calculated. The overall grade of a student's examination performance was calculated from the average of the total scores of both medical examiners.

#### 2.6. Qualitative data collection of participants’ experiences

The feasibility of the new M3 examination on outpatients was qualitatively analysed on the basis of the participants’ experiences described in the debriefings. Students and medical examiners participated in an approximately 30-minute group discussion with the study director directly after the examination. This was recorded in written from. The following aspects were covered:

- What should be considered when patients are pre-selected for examination by the teaching physician?
- How do the students assess the feasibility of the individual examination steps in terms of content and time?
- Are medical examiners and examiners from another health profession able to assess student examination performance using standardised evaluation forms?
- What are the implications of conducting the examination in the teaching practice on the day of the examination, and what premises are required for the examination?

Participating patients were interviewed in guided semi-structured interviews about their experience of the examination venue and procedure and their perception of the medical examiners during the examination (see attachment 1 ). The interviews were recorded, transcribed, and subjected to qualitative content analysis according to Mayring using MAXQDA 2020 Analytics Pro [13].

Furthermore, a quantitative estimate was made of how many general medical testing practices in Germany are required to conduct the new M3 examination with outpatients in order to implement the examination throughout Germany.

## 3. Results

### 3.1. Pre-selection and information of patients for examination 

The participating general practitioners selected mostly older patients with chronic diseases who were predominantly treated in the family practice. This enabled the students to inquire about disease-specific aspects in addition to general information when they talk with the patients, to survey the individual handling of the existing diseases and health competencies, and to specifically record disease-related findings in the comprehensive examination.

#### 3.2. Feasibility of the individual examination steps in terms of content and timing

All participating students indicated that the competencies for conducting a medical history and examination, intraprofessional handover, and preparing a medical report had been taught during their studies and appeared to be readily applicable in the pilot examination. In contrast, their prior knowledge and competencies for a case-based scientific question, interprofessional handover, and writing a patient-directed report varied. The examination step for planning ambulatory care management was predominantly familiar to eleven of 14 participating students who had completed the PJ elective in general medicine. 

#### 3.3. Performance evaluations by the examiners

**Figure 1:**
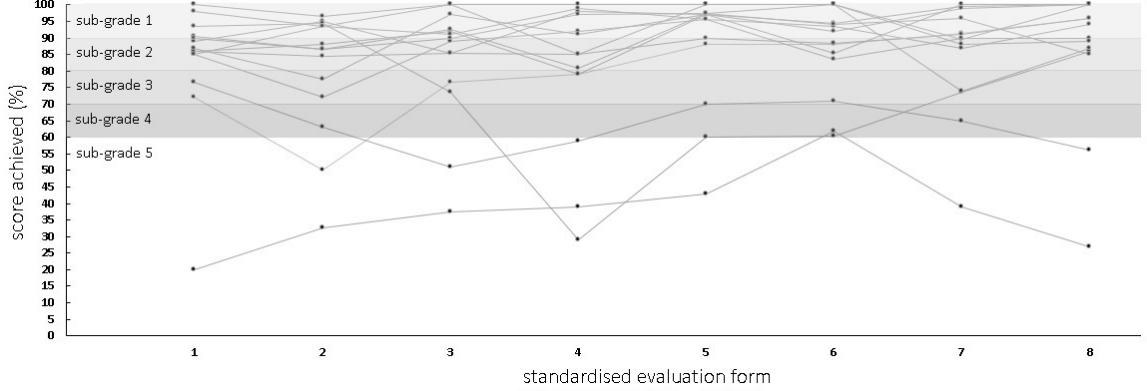
Sub-scores determined using standardised scoring sheets and associated percentile ranks of individual examinees (linked with lines) in the examination sub-steps of history (1), physical examination (2), intraprofessional handover (3), scientific question (4), care management proposal (5), interprofessional handover (6), evidence-based patient report (7), and a patient-directed report (8).

All examiners were able to evaluate the performance for each subtask, judged the items to be relevant in the exam debriefing, and judged the anchor criteria to the items to be helpful. The 14 participating students achieved overall scores ranging from “very good” (6x), “good” (4x), “satisfactory” (2x), and “sufficient” (1x) to “insufficient” (1x). Figure 1 (Fig. 1) illustrates the range of results achieved by the individual examinees per examination step. They ranged from 20% to 100% of the achievable points. In the debriefing, the examiners expressed a desire for detailed examiner training with provision of training materials to gain more confidence in evaluating student performance. The medical assistants evaluated the interprofessional transfer of students with similar results as the medical examiners.

#### 3.4. Impact of the test performance on practice operations on examination day and spatial requirements for the test practice

For the medical examiners, the examination time per student excluding any preparation was 2.25 hours. The second student's examination began 75 minutes after the that of the first student. The medical examiners examined both students alternating. With a break of 30 minutes for the examination participants, the total duration of the examination for the examiners was approximately 7 hours and 10 minutes. For the main examiner, this meant that other practical activities were largely impossible during this period. On condition of an appropriate fee for their work as examiners, the main examiners agreed to work as examiners in their practice approximately twice per year.

Several practice rooms were required for the examination. The medical history was taken and a comprehensive physical examination of the patient was conducted under the supervision of the main examiner and co-examiner in a treatment room. The results of the subsequent sections of the examination were also presented to the examiners in this room. In addition, a room with a computer with Internet access was required for each student to complete the scientific question (step 4) and to prepare a multisectoral care management plan (step 5) and to write the reports (steps 7 and 8).

#### 3.5. Reflections of the participating patients on the examination process and examination location 

The examination process was perceived by the patients as “very orderly, very well structured” (Patient 7 [P7]). At the same time, their statements also reflected the level of performance of the respective students in their interaction with the patient. One patient was very satisfied with the examination: “That's how I imagined it that she would examine me from top to bottom” [P3], while another described the course of the conversation critically: “Well, there was just this smoothness ... lacking” [P2]. The interviewed patients had an extremely positive perception of the fact that the pilot examination took place in the practice with the participation of their general practitioner (GP). The familiarity with the examination location and environment and the pre-existing trusting relationship with their treating GP, gave patients confidence to participate in the pilot examination and to engage with their role as an examination patient, which was new for most of them. They commented: “I knew this practice. And I thought that was good. Yes, it was not at all disconcerting for me” [P7] and “The location ... is familiar and that is reassuring. To know that one has been here before and is familiar with the immediate surroundings. This is no doubt important ...” [P13]. 

#### 3.6. Quantitative effort estimation at the national level

For the purpose of estimating the effort at the national level, we assume that examiners are willing to participate in examiner training and to conduct two appropriately remunerated examinations per year. With approximately 10,000 graduates per year [14], this means that 2500 examination practices in Germany are required in which a total of 5000 examinations with an examination duration of approximately 7 hours each are conducted annually. For each university, this means that 25 examination practices per one hundred graduates together with persons who coordinate the examination practices are required for the new examination format to be feasible.

## 4. Discussion

We conducted a feasibility study in general medical PJ teaching practices at several university locations. We showed by example that the new oral practical examination with outpatients can in principle be carried out successfully. All steps of the examination were performed by the participating students in terms of content and within the given time and organizational framework. The students assessed the tasks as largely achievable. In particular, competencies in communication, performing physical examinations, and preparing a treatment plan were taught in detail during the course of study. The teaching of scientific and interprofessional competencies and the skills for writing medical reports profited even more in the course of study, according to the assessment of the participating students. The examiners were able to assess the examination performance using standardised evaluation forms and suggested final adjustments for the examination. The participating patients appreciated the examination process and gained insight into the level of training and competencies of the examined students.

As far as we can ascertain from the international literature, comparable tests with real patients in a GP setting have only been established in a few countries to date, for example in the medical school in Leicester, UK [15]. General medicine has been one of the main examination subjects in Norway [16] since the 1990s. Another example is a two-hour examination with general practice patients that has been conducted in family practices connected with the University of Tromso, Sweden, since 2004. This was evaluated over three years [17]. Students were examined by a general practitioner and a clinician examiner during a patient consultation and in a subsequent examination interview. This examination also included the history taking, examination, scientific questioning, and comprehensive healthcare planning substeps, but did not include interprofessional handover and report writing.

The IMPP’s new examination format in Germany represents a competence-oriented further development of the oral practical examination at the patient's bedside that is at present established in the final medical examination [https://www.gesetze-im-internet.de/_appro_2002/BJNR240500002.html]. It adopts the concept of workplace-based review of professional activities that can be entrusted to the trainee or student, including interprofessional communication and the formulation of reports that can be understood by physicians and patients [6], [18], [19]. With its implementation in the general medical practice, it furthermore enables a high degree of practical proximity and also an interdisciplinary exchange between general practitioners and clinically active examiners. The general medical institutes of the medical faculties in Germany already cooperate with numerous teaching practices that help implement and conduct the PJ. Existing concepts and experience from model projects can be used to recruit and accredit additional teaching and new examination practices [20], [21].

The oral-practical pilot examinations were conducted with outpatients in the same way as they would take place in real examinations. In contrast to the future medical final examination, the students were still in the PJ, had completed their studies according to the specifications of the currently valid ÄApprO and only partially completed the PJ in general medicine. They were examined in practices with which they were familiar in part through the PJ, and had not prepared for this examination in particular. 

Implementation of the new final examination is dependent on the restructuring of medical studies that is intended in the Master Plan 2020. This includes competency-based training, longitudinal curricula for communication and general medicine, more specific teaching of interprofessional and scientific competencies, and a mandatory outpatient quarter [7]. The students should also be enabled to receive workplace-based formative feedback on their current performance status as part of their PJ. For this purpose, the standardised evaluation forms of the pilot examinations provide specific suggestions for structuring such feedback. Formative feedback can be given for each of the eight examination steps. It is possible to integrate formative feedback into PJ teaching with a time requirement of approximately 10 minutes for feedback on the individual subtasks of the overall examination.

In the pilot examinations, the study director provided instruction on the examination process and the procedure for evaluating examination performance. Implementation beyond the pilot phase requires examiner training, provision of materials for training and documentation of the examination performance, and also appropriate compensation for examiner time and effort per exam day. 

The pilot study found that general medical teaching practices have patients who were highly motivated to participate in the examination. This allowed students to apply and demonstrate their knowledge and skills to the examiners. Based on the experience of the pilot examinations, recommendations can be developed for future testing practices regarding patients who are appropriate for testing. Materials for the information of interested patients can also be created and provided. In addition, privacy requirements [22] and other legal aspects such as the insurance coverage of the students that are to be tested and of the participating patients must be taken into account.

## 5. Conclusions

The newly designed oral-practical examination with outpatients was successfully carried out as part of a pilot study. Overall, the new examination concept represents a challenge for all involved. It will take several years from the implementation of the new ÄApprO, before examinations like this need to be established. This means that we have the opportunity to develop the required structures together with organisational and examiner competencies. For examiner qualification, the IMPP is developing a training concept. Evaluation forms already exist in advanced pilot versions, which will be further adapted and can be tested for validity and reliability in further pilot studies. The new final examination enables us to reflect the vision outlined in the Master Plan of a more practical, more scientific and patient-oriented education of medical students in the final examination and to design it more realistic and fair.

## Notes

### Gender-sensitive language

We predominantly use the masculine form for persons, but all genders are always meant equally.

#### Ethics vote

Positive votes for the study have been received from the following ethics committees:

University Medical Center Göttingen (26/1/20), Medical Association of Lower Saxony (027/2020), Medical Association of Westphalia-Lippe (2020-136-b-S), Philipps University Marburg (35/20). The study was registered in the German Register of Clinical Trials under the number 00020565.

## Acknowledgements

The authors would like to thank the patients involved in the study, the resident PJ teaching physicians Dr. U. Annweiler (Waake), Dr. R. Beverungen (Höxter), Mr. M. Eckert (Herzberg), Dr. A. Hähnel (Göttingen), Dr. W. Keske (Göttingen), Dr. D. Ladwig (Homberg), Dr. M. Lang (Göttingen), Dr. M. Schünemann (Nörten-Hardenberg), and Dr. K. Wetzel (Göttingen), the colleagues PD Dr. C. Brünahl, Prof. Dr. A. Oksche and Mr. U. Scherer of the IMPP, the workshop participants, and the colleagues of the University Medical Center Göttingen for their cooperation and suggestions for the further development of the examination.

## Competing interests

The authors declare that they have no competing interests. 

## Supplementary Material

Guidelines for the semi-structured interview with patients in the pilot audit. These are excerpts from a more comprehensive interview about the experience of patient-centredness in the examination.

## Figures and Tables

**Table 1:**
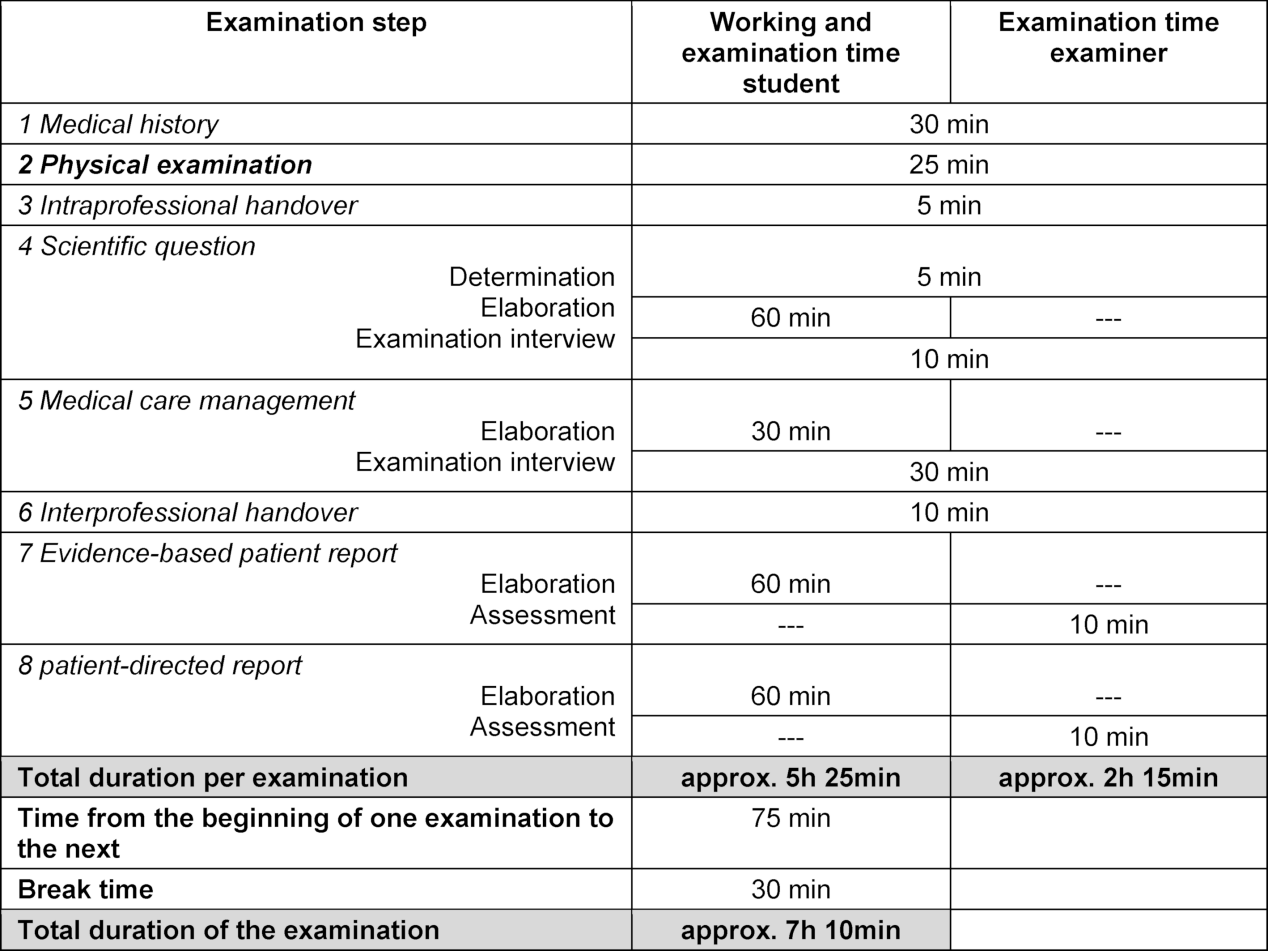
Overview of exam steps and timeframes for students and examiners
