# Supplementary material for: Implementation of a uniform nationwide medical licensing examination in general practice. A feasibility study
Source: GMS J Med Educ. 2021 Jun 15;38(5):Doc96. doi: 10.3205/zma001492 (PMC8256129; doi:10.3205/zma001492)
Supplement: Guidelines for the semi-structured interview with patients in the pilot audit. These are excerpts from a more comprehensive interview about the experience of patient-centredness in the examination. [file JME-38-5-96-s-001.pdf]

Attachment 1: Guidelines for the semi-structured interview with patients in the pilot study. These are excerpts from a more comprehensive interview about the experience of patient-centredness in the examination.

Thank you for agreeing to assist us in our study by conducting this interview. You had a detailed conversation with the student in the examination. I would like to ask you a few questions about how you perceived the student in this situation and how the conversation went.

How did you experience the conversation?

> *If applicable, sub-question: How did you perceive the atmosphere of the conversation?*

I would now like to ask two more questions about the examination environment and design.

How did you experience the examination venue and the examination process?

How did you perceive the medical examiners during the examination?

Is there anything else you would like to add or share that you haven't told me yet, but that is important to you in connection with the examination you experienced?

Thank you very much for the interview and for your cooperation.

Attachment 1 to: Demmer I, Selgert L, Altiner A, Baum E, Becker A, Schmittdiel L, Streitlein-Böhme I, Michiels-Corsten M, Zutz S, Hummers E, Jünger J. *Implementation of a uniform nationwide licensing examination for medical students in general practice.* A feasibility study. GMS J Med Educ. 2021;38(5):Doc96. DOI: 10.3205/zma001492
